# Endoscopic variceal obturation and retrograde transvenous obliteration for acute gastric cardiofundal variceal bleeding in liver cirrhosis

**DOI:** 10.1186/s12876-022-02428-1

**Published:** 2022-07-26

**Authors:** Han Ah Lee, Jungwon Kwak, Sung Bum Cho, Young-Sun Lee, Young Kul Jung, Ji Hoon Kim, Seung Up Kim, Hyonggin An, Hyung Joon Yim, Jong Eun Yeon, Yeon Seok Seo

**Affiliations:** 1 Departments of Internal Medicine, Ewha Womans University College of Medicine, Seoul, Korea; 2 Department of Radiology, Korea University College of Medicine, Seoul, Korea; 3 Department of Internal Medicine, Korea University College of Medicine, Seoul, Korea; 4 Department of Internal Medicine, Yonsei University College of Medicine, Seoul, Korea; 5 Yonsei Liver Center, Severance Hospital, Seoul, Korea; 6 Department of Biostatistics, Korea University College of Medicine, Seoul, Korea

**Keywords:** Rebleeding, Prevention, Balloon-occluded retrograde transvenous obliteration, Vascular plug-assisted retrograde transvenous obliteration, Portal hypertension

## Abstract

**Background/Aims:**

We retrospectively compared the effect of endoscopic variceal obturation (EVO) and retrograde transvenous obliteration (RTO) in acute cardiofundal variceal bleeding.

**Methods:**

Patients with acute cardiofundal variceal bleeding treated with EVO or RTO at two hospitals were included.

**Results:**

Ninety patients treated with EVO and 86 treated with RTO were analyzed. The mean model for end-stage liver disease score was significantly higher in EVO group than in RTO group (13.5 vs. 11.7, *P* = 0.016). The bleeding control rates were high (97.8% vs. 96.5%), and the treatment-related complication rates were low in both EVO and RTO groups (2.2% vs. 3.5%). During the median follow-up of 18.0 months, gastric variceal (GV) and esophageal variceal rebleeding occurred in 34 (19.3%) and 7 (4.0%) patients, respectively. The all-variceal rebleeding rates were comparable between EVO and RTO groups (32.4% vs. 20.8% at 2-year, *P* = 0.150), while the GV rebleeding rate was significantly higher in EVO group than in RTO group (32.4% vs. 12.8% at 2-year, *P* = 0.003). On propensity score-matched analysis (71 patients in EVO vs. 71 patients in RTO group), both all-variceal and GV rebleeding rates were significantly higher in EVO group than in RTO group (all *P* < 0.05). In Cox regression analysis, EVO (vs. RTO) was the only significant predictor of higher GV rebleeding risk (hazard ratio 3.132, *P* = 0.005). The mortality rates were similar between two groups (*P* = 0.597).

**Conclusions:**

Both EVO and RTO effectively controlled acute cardiofundal variceal bleeding. RTO was superior to EVO in preventing all-variceal and GV rebleeding after treatment, with similar survival outcomes.

**Supplementary Information:**

The online version contains supplementary material available at 10.1186/s12876-022-02428-1.

## Introduction

Gastric varices (GVs) are enlarged submucosal veins of the stomach that are present in approximately 20% of patients with liver cirrhosis [1]. Bleeding from GVs is less frequent than from esophageal varices (EVs), with a bleeding rate of 25% over 2 years [1]. However, GVs that bleed are mostly large and have high blood flow, which can result in severe bleeding [1–4]. Moreover, rebleeding and mortality rates are also higher in GVs than in EVs [1, 5, 6].

According to their location, gastroesophageal varices (GOV) 2 and isolated GV (IGV) 1 varices are usually classified as cardiofundal varices [7]. Treatment of cardiofundal variceal bleeding can be difficult, since cardiofundal varices are larger and have more complicated blood circulation than GOV1s [8–10]. Accompanied collateral shunts are other barriers to achieving a complete cure of cardiofundal varices [11].

Current guidelines recommend endoscopic variceal obturation (EVO) as one of the treatment options for acute GV bleeding [7, 12, 13]. The rate of hemostasis after EVO has been reported to be as high as 91–100%; however, the rebleeding rate from GVs after EVO remains at 3.6–41.0% [14–18].

Recently, retrograde transvenous obliteration (RTO), including balloon-occluded (BRTO) and vascular plug-assisted RTO (PARTO), have been considered as the treatment options for acute cardiofundal variceal bleeding. High hemostasis rates (> 90%) and low rebleeding rate (0–7.43%) have been reported in patients treated with BRTO in acute GV bleeding [19–21]. Further, PARTO showed high technical and clinical success rates and no rebleeding events in patients with GV bleeding [22–24].

However, to date, an optimal treatment modality for acute cardiofundal variceal bleeding has not been confirmed. Accordingly, we compared the efficacy and safety of EVO and RTO for acute cardiofundal variceal bleeding in patients with cirrhosis.

## Materials and methods

### Study population

**Fig. 1:**
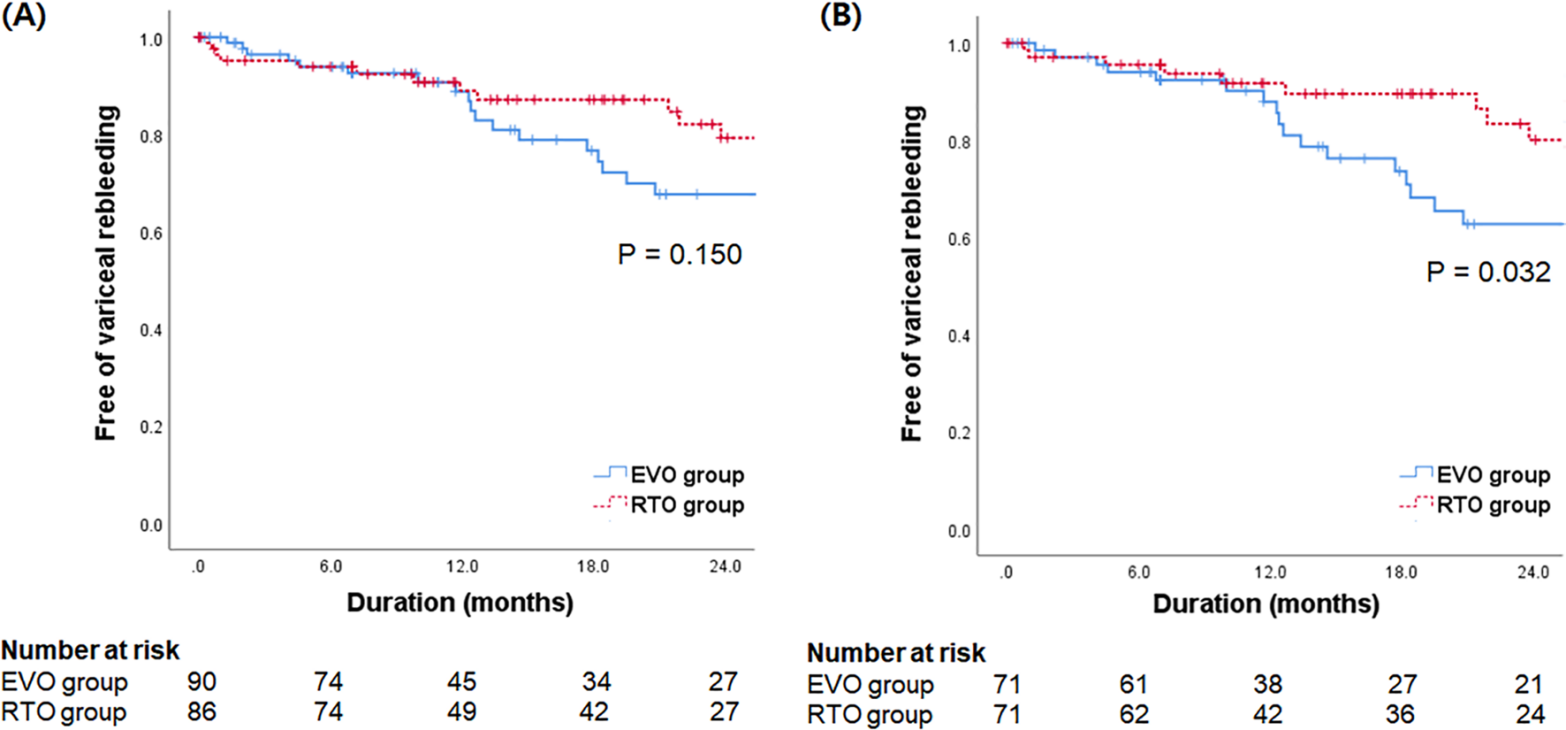
Cumulative variceal rebleeding rate according to the type of treatment in all patients (**A**) and in patients balanced by propensity score matching (**B**)

Patients with acute cardiofundal variceal bleeding who were treated with EVO or RTO between March 2006 and November 2018 at the Korea University Anam Hospital and Yonsei University Severance Hospital were retrospectively included (Fig. 1). The exclusion criteria were as follows: (a) age < 18 years, (b) insufficient follow-up period (less than 6 months), (c) previous treatment with EVs or GVs, (d) non-cirrhotic portal hypertension, (e) portal vein thrombosis, (f) advanced malignancy including hepatocellular carcinoma (HCC), and (g) history of organ transplant.

### Definition

Liver cirrhosis was diagnosed either clinically or histologically when typical ultrasonographic findings were present and consistent with a low platelet count (< 100,000/μL) or overt complications of liver cirrhosis [25]. Acute cardiofundal variceal bleeding was diagnosed if the following were present on esophagastroduodenoscopy (EGD) [26, 27]: (a) active blood spurting or oozing from cardiofundal varices; (b) blood clots or white nipples on the surfaces of cardiofundal varices; (c) blood in the stomach without a potential bleeding cause other than cardiofundal varices.

### Treatments

When acute cardiofundal variceal bleeding was suspected, vasoactive drugs such as terlipressin or somatostatin were administered, followed by diagnostic EGD within 12 h. When acute cardiofundal variceal bleeding was detected on EGD, EVO or RTO was performed within 6 h, depending on the presence of a gastrorenal shunt, availability of resources or expertise for RTO, and the clinician’s decision. When the clinician decided to perform RTO, endoscopists did not perform any endoscopic treatment. Patients who underwent EVO were classified into the EVO group, and those who underwent RTO, including BRTO or PARTO, were classified into the RTO group. Detailed procedures of EVO, BRTO, and PARTO are described in Additional file 1.

### Outcomes

The primary outcomes were all-variceal and GV rebleeding. Rebleeding was defined as recurrent bleeding after an absence of bleeding for at least 5 days following resolution of acute GV bleeding [28]. The diagnosis of variceal rebleeding was the same as that for acute variceal bleeding. The secondary outcomes were bleeding control, treatment-related complications, and mortality. Patients were followed up until death, liver transplantation, or loss to follow-up.

### Statistical analysis

Demographic and laboratory data are presented as mean ± standard deviation for continuous variables and numbers with percentages for categorical variables. Categorical and quantitative variables of the groups were compared using the chi-square test and Student's *t*-test, respectively.

To minimize the potential bias according to the different baseline characteristics between the EVO and RTO groups, propensity score matching (PSM) was calculated by fitting a logistic regression model that included the following variables in both the EVO and RTO cohorts: age, sex, diabetes, HCC, type of varices, size of EVs, hemoglobin, platelet count, INR, serum levels of albumin, total bilirubin and ALT, and model for end-stage liver disease (MELD) score. A 1:1 ratio PSM was performed using the nearest neighbor method.

Variceal rebleeding and mortality rates were estimated using the Kaplan–Meier method and compared using the log-rank test. The data of patients that died, received a liver transplantation, or were lost to follow-up were censored. Independent predictors for variceal rebleeding and mortality were evaluated using the Cox proportional hazard regression analysis. All statistical analyses were performed using the Statistical Package for the Social Sciences version 25.0 software (International Business Machines Corp.).

## Results

### Patient characteristics

Among the 307 eligible patients, a total of 176 patients were finally selected for statistical analyses (90 [51.1%] in the EVO group and 86 [48.9%] in the RTO group) (Additional file 2: Fig. S1). In the RTO group, 45 (52.3%) and 41 (47.7%) patients were treated with BRTO and PARTO, respectively. The baseline characteristics of the study population are shown in Table 1. Ninety-nine (56.3%) patients had GOV2, and 77 (43.7%) patients had IGV1. The mean MELD score was 12.6. Beta-blockers were administered to 52 (29.5%) patients after bleeding control was achieved.

**Table 1:** Baseline characteristics of all patients

	All patients (n = 176)	EVO group (n = 90, 51.1%)	RTO group (n = 86, 48.9%)	*P* value
Age	60.3 ± 11.7	60.5 ± 11.9	59.5 ± 11.7	0.704
Male, n (%)	125 (71.0)	69 (76.7)	56 (65.1)	0.091
Etiology, n (%)				0.395
Hepatitis B virus	50 (28.4)	22 (24.4)	28 (32.6)	
Hepatitis C virus	14 (8.0)	7 (7.8)	7 (8.1)	
Alcohol	83 (47.2)	48 (53.3)	35 (40.7)	
Other	29 (16.5)	13 (14.4)	16 (18.6)	
Diabetes, n (%)	50 (28.4)	24 (26.4)	26 (30.2)	0.298
Hepatocellular carcinoma, n (%)	50 (28.4)	24 (26.7)	26 (30.2)	0.600
Type of varices, n (%)				0.470
GOV2	99 (56.3)	53 (58.9)	46 (53.5)	
IGV1	77 (43.7)	37 (41.1)	40 (46.5)	
Size of esophageal varices, n (%)				0.244
F0–F1	102 (58.0)	47 (52.2)	55 (64.0)	
F2–F3	74 (42.0)	43 (47.8)	31 (36.0)	
Hemoglobin, g/dL	9.0 ± 5.3	8.9 ± 2.2	9.1 ± 2.4	0.504
Platelet count, × 10^9^/L	103.1 ± 52.6	106.0 ± 45.5	100.4 ± 59.7	0.935
INR	1.43 ± 0.32	1.44 ± 0.36	1.41 ± 0.71	0.362
Alanine aminotransferase, IU/L	38.5 ± 49.7	42.1 ± 60.9	35.1 ± 34.5	0.418
Total bilirubin, mg/dL	2.1 ± 3.2	2.3 ± 3.7	1.8 ± 2.5	0.154
Serum albumin, g/dL	2.9 ± 0.2	2.9 ± 0.5	3.0 ± 0.5	0.274
MELD score	12.6 ± 4.7	13.5 ± 4.5	11.7 ± 4.8	0.016
Beta-blockers, n (%)	52 (29.5)	11 (12.2)	41 (47.7)	< 0.001

Variables are expressed as mean ± standard deviation or n (%)

*EVO* endoscopic variceal obturation, *RTO* retrograde transvenous obliteration, *GOV2* gastroesophageal varices type 2, *IGV1* isolated gastric varices type 1, *INR* international normalized ratio, *MELD*, model for end-stage liver disease

### Comparison between the EVO and RTO group

Baseline characteristics were statistically similar between the EVO and RTO groups (all *P* > 0.05), except for a significantly higher MELD score in the EVO group than in the RTO group (mean 13.5 vs. 11.7, *P* = 0.016) (Table 1). The proportions of GOV2 (58.9% vs. 53.5%) and IGV1 (41.1% vs. 46.5%) were statistically similar between the EVO and RTO groups (*P* = 0.470). Seventy patients (77.8%) in EVO group had a gastrorenal shunt feasible for RTO procedure.

The proportion of patients who were treated with beta-blockers after bleeding control was significantly higher in the RTO group than in the EVO group (47.7% vs. 12.2%, *P* < 0.001). The proportion of patients treated with propranolol and carvedilol was 75.0% and 25.0% in the EVO group and 73.2% and 26.8% in the RTO group, respectively (*P* = 0.735). The mean doses of both propranolol (48.9 mg vs. 51.7 mg, *P* = 0.807) and carvedilol (12.5 mg vs. 14.7 mg, *P* = 0.567) were statistically similar between the two groups.

### Treatment outcomes

Bleeding was successfully controlled in 171 patients (97.2%). The bleeding control rate was similar between the two groups (97.8% in the EVO group vs. 96.5% in the RTO group, *P* = 0.613) (Additional file 2: Table S1). In 5 patients who failed to achieve bleeding control, transjugular intrahepatic portosystemic shunt were performed. Technical success was achieved in all patients in the EVO and RTO groups. Among patients in the EVO group, 48 patients achieved obliteration of the GV with one session. The other 42 patients underwent additional endoscopic intervention within 1 week of the initial treatment. The mean number of performed EVO sessions was 1.5 ± 1.0, and the mean volume of cyanoacrylate mixture used in each patient was 4.4 ± 2.7 mL.

Treatment-related complications were investigated. Two patients in the EVO group and one patient in the RTO group had worsening ascites, and two patients in the RTO group developed hepatic encephalopathy. No systemic embolization or thrombus developed in either group.

### Change of esophageal varices after the treatment

After the treatment for acute cardiofundal variceal bleeding, worsening of EVs was found in 12 (14.8%) patients in the EVO group and 24 (27.9%) patients in the RTO group (*P* < 0.001). Among patients with F2–F3 EVs after the treatment for acute cardiofundal variceal bleeding, 25 of 36 (69.4%) in the EVO group and 25 of 32 (78.1%) in the RTO group underwent endoscopic variceal ligation (EVL) as a secondary prevention of EV bleeding.

### All-variceal rebleeding

During the median follow-up period of 18.0 (interquartile range, 7.0–38.9) months, all-variceal rebleeding occurred in 41 (23.3%) patients (26 in the EVO group and 15 in the RTO group). The most common type of variceal rebleeding was GV bleeding (n = 34), followed by EV bleeding (n = 7). All EV rebleeding developed after RTO, and most cases (6 out of 7) did not receive EVL after RTO.

All-variceal rebleeding rates at 6, 12, 18, and 24 months after treatment were 6.1%, 11.1%, 17.9%, and 26.3%, respectively (Additional file 2: Table S1). The corresponding rates in the EVO and RTO groups were statistically similar (6.1%, 11.2%, 23.4%, and 32.4% vs. 6.1%, 11.1%, 12.9%, and 20.8%, respectively, *P* = 0.150 by log-rank test) (Fig. 1A). There was no significant difference in all-variceal rebleeding rates between the BRTO and PARTO groups (*P* = 0.891 by log-rank test). In the Cox regression analysis, no significant predictor of all-variceal rebleeding was found (Table 2).

**Table 2:** Predictors for variceal rebleeding

Variables	Rating	Univariate analysis for variceal bleeding			Univariate analysis for gastric variceal bleeding		
		Hazard ratio	95% CI	*P* value	Hazard ratio	95% CI	*P* value
Age	Years	0.997	0.969–1.025	0.814	1.000	0.970–1.032	0.992
Sex	0 = women; 1 = men	1.384	0.678–2.825	0.373	1.447	0.654–3.198	0.362
Diabetes	0 = no; 1 = yes	1.019	0.524–1.982	0.955	1.060	0.513–2.192	0.874
Etiology	0 = other; 1 = alcohol	1.195	0.644–2.219	0.573	1.242	0.630–2.450	0.531
Size of esophageal varices	0 = F0, F1; 1 = F2, F3	1.131	0.595–2.149	0.707	1.091	0.536–2.222	0.809
Hemoglobin	g/dL	0.977	0.869–1098	0.695	0.976	0.859–1.109	0.707
Platelet count	× 10^9^/L	1.003	0.998–1.009	0.261	1.002	0.995–1.008	0.628
INR		0.732	0.233–2.301	0.593	0.944	0.348–2.563	0.910
Alanine aminotransferase	IU/L	1.002	0.995–1.008	0.642	1.002	0.994–1.009	0.679
Total bilirubin	mg/dL	1.017	0.942–1.098	0.662	1.026	0.948–1.110	0.520
Serum albumin	g/dL	1.106	0.590–2.073	0.754	0.808	0.402–1.627	0.551
MELD score		1.025	0.952–1.103	0.515	1.049	0.973–1.131	0.213
Type of varices	0 = GOV2; 1 = IGV1	1.190	0.6041–2.207	0.581	1.008	0.511–1.990	0.981
Type of treatment	0 = RTO; 1 = EVO	1.557	0.816–2.970	0.179	3.132	1.408–6.970	0.005
Beta-blocker	0 = no; 1 = yes	0.879	0.430–1.798	0.724	0.994	0.463–2.136	0.988
Post EVL	0 = no; 1 = yes	0.652	0.324–1.310	0.229	0.760	0.360–1.606	0.473

*IGV1* isolated gastric varices type 1, *GOV2* gastroesophageal varices type 2, *INR* international normalized ratio, *MELD* model for end-stage liver disease, *EVO* endoscopic variceal obturation, *RTO* retrograde transvenous obliteration, *EVL* endoscopic variceal ligation

We additionally evaluated whether this result was reproducible after PSM, and the clinical characteristics of patients balanced by PSM (71 patients in EVO group vs. 71 patients in RTO group) are presented in Additional file 2: Table S2. On PSM analysis, all-variceal rebleeding rate was significantly higher in the EVO group than in the RTO group (6.0%, 12.1%, 26.4%, and 37.3% vs. 4.5%, 8.3%, 10.5%, and 20.0%, respectively, *P* = 0.032 by log-rank test) (Fig. 1B, Additional file 2: Table S3).

### GV rebleeding

**Fig. 2:**
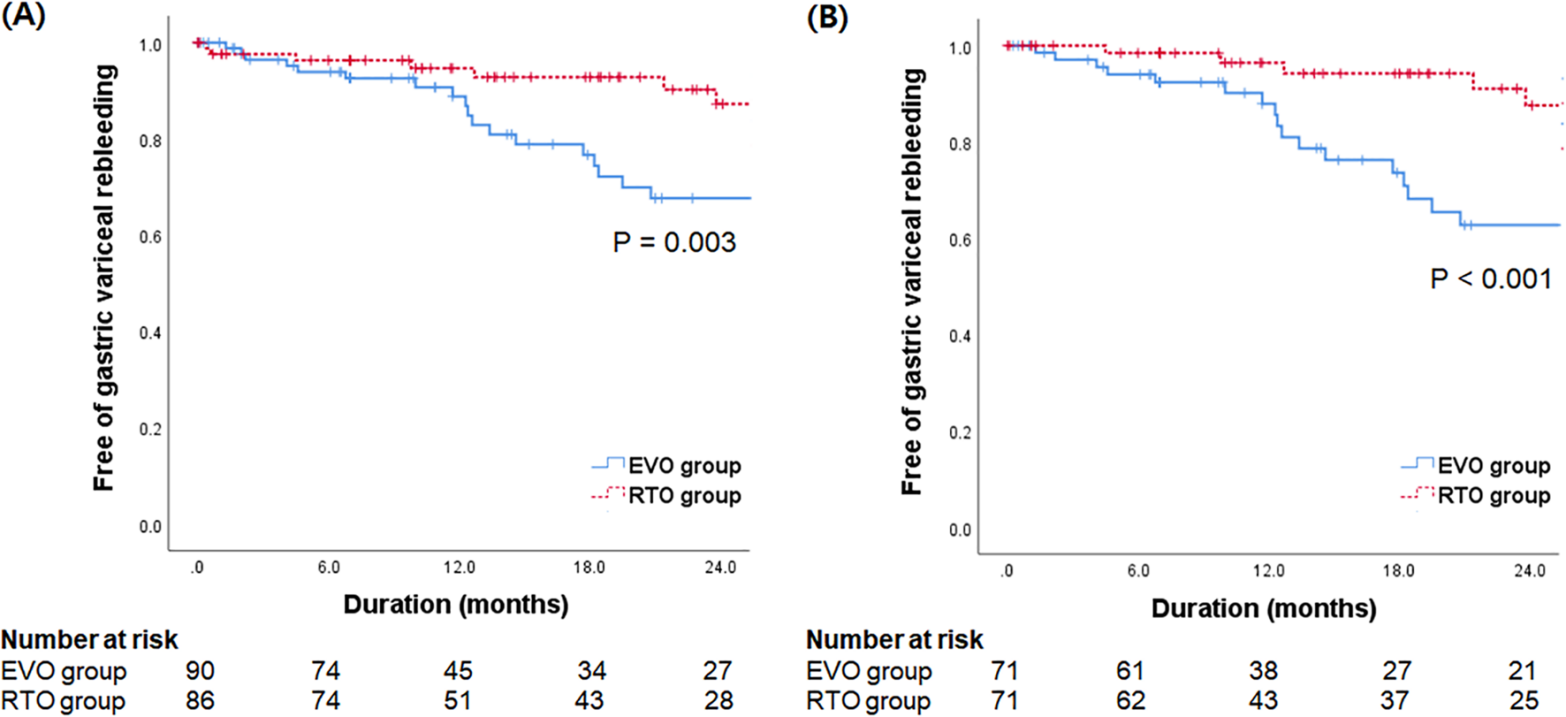
Cumulative gastric variceal rebleeding rate according to the type of treatment in all patients (**A**) and in patients balanced by propensity score matching (**B**)

GV rebleeding was analyzed separately. GV rebleeding rates at 6, 12, 18, and 24 months after treatment were 4.9%, 8.3%, 15.2%, and 22.5%, respectively (Additional file 2: Table S1). The corresponding rates in the EVO group were significantly higher than those in the RTO group (6.1%, 11.2%, 23.4%, and 32.4% vs. 3.7%, 5.4%, 7.2%, and 12.8%, respectively, *P* = 0.003 by log-rank test) (Fig. 2A). No significant difference in GV rebleeding rate was observed between the BRTO and PARTO groups (*P* = 0.838 by log-rank test). In the Cox regression analysis, EVO treatment (vs. RTO) was the only significant predictor of higher risk of GV rebleeding (hazard ratio [HR] = 3.132, 95% confidence interval [CI] 1.408–6.970, *P* = 0.005) (Table 2).

On PSM analysis, GV rebleeding rate was significantly higher in the EVO group than in the RTO group (6.0%, 12.1%, 26.4%, and 37.3% vs. 1.6%, 3.6%, 5.8%, and 12.5%, respectively, *P* < 0.001 by log-rank test) (Fig. 2B, Additional file 2: Table S3).

### Mortality

**Fig. 3:**
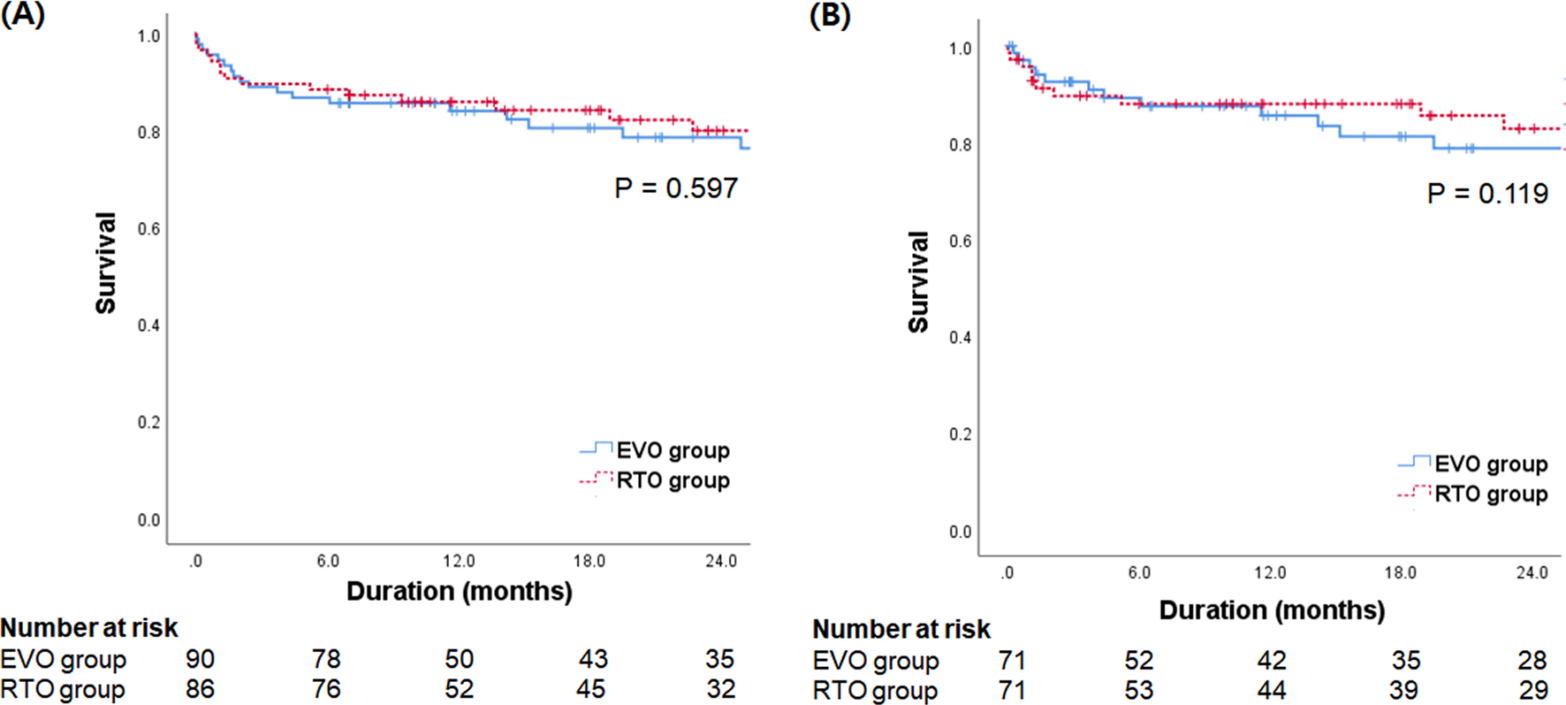
Cumulative transplantation-free survival rates according to the type of treatment in all patients (**A**) and in patients balanced by propensity score matching (**B**)

During the follow-up period, 40 patients died (21 in the EVO group and 19 in the RTO group). The causes of death were variceal bleeding (27.5%), infection (30.0%), or liver failure (42.5%). Six patients received liver transplantation (three patients in the EVO group and three patients in the RTO group). The cumulative transplantation-free survival rates at 6, 12, 18, and 24 months after treatment were 87.5%, 84.8%, 82.2%, and 79.1%, respectively (Additional file 2: Table S1). No significant difference in transplantation-free survival rates was observed between the EVO group (86.7%, 83.9%, 80.4%, and 78.4%) and the RTO group (88.4%, 85.8%, 84.1%, and 79.9%, *P* = 0.597 by log-rank test) (Fig. 3A). In the Cox regression analysis, higher MELD score was the only independent predictor for higher risk of mortality (HR = 1.089, 95% CI 1.030–1.151, *P* = 0.002), whereas EVO (vs. RTO) was not (*P* = 0.598) (Table 3).

**Table 3:** Predictors for mortality

Variables	Rating	Univariate analysis		
		Hazard ratio	95% CI	*P* value
Age	Years	1.005	0.979–1.031	0.721
Sex	0 = women; 1 = men	1.418	0.702–2.865	0.330
Diabetes	0 = no; 1 = yes	1.039	0.559–1.932	0.904
Etiology	0 = other; 1 = alcohol	1.750	0.959–3.195	0.068
Hemoglobin	g/dL	1.003	0.896–1.124	0.955
Platelet count	× 10^9^/L	0.998	0.992–1.005	0.613
INR		1.268	0.860–1.871	0.231
Alanine aminotransferase	IU/L	1.001	0.995–1.007	0.748
Total bilirubin	mg/dL	1.050	0.992–1.110	0.090
Serum albumin	g/dL	0.674	0.363–1.252	0.212
MELD score		1.089	1.030–1.151	0.002
Type of varices	0 = GOV2; 1 = IGV1	1.696	0.932–3.085	0.084
Type of treatment	0 = RTO; 1 = EVO	1.174	0.648–2.127	0.598
Beta-blocker	0 = no; 1 = yes	1.381	0.741–2.574	0.309
Post EVL	0 = no; 1 = yes	1.570	0.859–2.866	0.142

*IGV1* isolated gastric varices type 1, *GOV2* gastroesophageal varices type 2, *INR* international normalized ratio, *MELD* model for end-stage liver disease, *EVO* endoscopic variceal obturation, *RTO* retrograde transvenous obliteration, *EVL* endoscopic variceal ligation

On PSM analysis, no significant difference in transplantation-free survival rates was observed between the EVO group (86.7%, 83.9%, 80.4%, and 78.4%) and the RTO group (88.4%, 85.8%, 84.1%, and 79.9%, *P* = 0.119 by log-rank test) (Fig. 3B, Additional file 2: Table S3).

## Discussion

Currently, an optimal treatment for acute cardiofundal variceal bleeding has not been confirmed. In this study, we directly compared EVO with RTO for acute cardiofundal variceal bleeding in patients with cirrhosis and found that all-variceal rebleeding rates at 2 years were statistically similar between the two groups (*P* = 0.150). However, the GV rebleeding rate at 2 years was significantly higher in the EVO group than in the RTO group (*P* = 0.003), and EVO (vs. RTO) was the only predictor of higher risk of GV rebleeding. On PSM analysis, both all-variceal and GV rebleeding rates were significantly higher in the EVO group than in the RTO group (all *P* < 0.05). Finally, we found that both EVO and RTO were effective for bleeding control (> 96.5%) and had low complication rates (< 3.5%). No difference was observed in the mortality between the two groups.

This study has several important clinical implications. In the present study, 1- and 2-year all-variceal rebleeding rates were statistically similar between the two groups. However, when patients were analyzed for GV rebleeding, the EVO group had significantly higher 1- and 2-year GV rebleeding rates than those in the RTO group. The difference between all-variceal and GV rebleeding rates could be explained by the high EV rebleeding rate after RTO. Rebleeding from EVs developed in seven patients treated with RTO; 6 out of 7 did not receive EVL after RTO. In the present study, 14.8% of patients in the EVO group and 27.9% of patients in the RTO group developed worsening of EVs after bleeding control (*P* < 0.001). A recent randomized controlled study reported an EV worsening rate of 30% and 43.5% in the EVO and BRTO groups, respectively [29], and similar results have been frequently reported in previous studies [9, 30–33]. Because RTO completely obliterates the portosystemic shunts that supply GVs, worsening of portal hypertension and its complications have been widely observed [34, 35]. Thus, screening endoscopy and appropriate prophylaxis with EVL could decrease EV rebleeding after RTO [29].

To minimize the potential bias according to the differences in baseline characteristics between the EVO and RTO groups, particularly in MELD scores, PSM analysis was performed. On PSM analysis, both 1- and 2-year all-variceal and GV rebleeding rates were significantly higher in EVO group than in RTO group. To our knowledge, three studies directly compared EVO and BRTO in terms of GV bleeding, and all studies demonstrated the superiority of BRTO over EVO in preventing variceal rebleeding [27, 29, 36]. A retrospective study of cardiofundal variceal bleeding found lower rates of rebleeding following BRTO compared to EVO; however, 16/71 patients who underwent BRTO had simultaneous transjugular intrahepatic portosystemic shunts, which could improve portal hypertension and further decrease GV rebleeding [36]. Recently, a randomized controlled study compared EVO with BRTO for secondary prophylaxis of cardiofundal GV bleeding [29]. However, the number of patients was small (32 patients in EVO group vs. 32 patients in BRTO group). Additionally, 67.2% of patients were transferred patients who had recovered from a previous GV bleeding within 4 weeks. A prospective study also found a higher variceal rebleeding rate for EVO than for BRTO (71.4% vs. 15.4%); however, BRTO was performed only in patients without active bleeding [27].

The rates of recurrence and rebleeding of GVs after successful RTO are low, possibly because the injected sclerosing agent completely destroys the venous endothelium. [30, 31]. The higher rebleeding rate in patients treated with EVO may be related to incomplete impaction of cyanoacrylate, leading to less or delayed obturation of GVs and their feeding vessels. Additionally, the results of EVO vary according to the clinician’s experience. Therefore, clinicians should seek the best option for each patient based on the patient’s general condition and access to appropriate medical resources and expertise in clinical practice.

The one of most common causes of death after acute cardiofundal variceal bleeding was variceal rebleeding (27.5%), emphasizing the need for proper prevention of variceal rebleeding after bleeding control is achieved. There was no significant difference in transplantation-free survival between the EVO and RTO groups, and a higher MELD score was the only predictor of increased mortality, consistent with the results of previous studies [27, 29].

The major limitation of our study is its retrospective design, which could have resulted in selection bias. Apparantly, patients in the EVO group had significantly worse liver function than those in the RTO group. Therefore, we conducted robust PSM analysis with large number of variables to minimize potential bias. In addition, due to the small number of patients who received beta-blockers, whether adding beta-blockers, known to have beneficial effects in the prognosis of patients with liver cirrhosis, can reduce rebleeding or mortality has not been elucidated by the current results. Finally, this study included both BRTO and PARTO, which are different treatment modalities that use different sclerosing agents. Although no differences were observed in all-variceal and GV rebleeding rates between the two groups, these results are not conclusive due to the small number of patients in each group. Further randomized controlled studies with larger numbers of patients are needed to confirm the optimal treatment strategy for patients with acute cardiofundal variceal bleeding.

In conclusion, our study shows that both RTO and EVO are effective and safe methods, however, RTO is more effective than EVO in preventing all-variceal and GV rebleeding, with similar survival outcomes. The worsening of EVs after BRTO should be screened and managed appropriately. EVO could be another effective option for acute cardiofundal variceal bleeding, especially in a clinical setting that lacks resources or expertise for RTO.

## Supplementary Information

**Additional file 1.** Supplementary Note.

**Additional file 2. Supplementary Figure 1**. Flowchart of patients.

## Data Availability

The datasets are available from the corresponding author on reasonable request.

## Abbreviations

GVs: Gastric varices
EVs: Esophageal varices
GOV: Gastroesophageal varices
IGV: Isolated gastric varices
EVO: Endoscopic variceal obturation
RTO: Retrograde transvenous obliteration
BRTO: Balloon-occluded retrograde transvenous obliteration
PARTO: Vascular plug-assisted retrograde transvenous obliteration
HCC: Hepatocellular carcinoma
EGD: Esophagastroduodenoscopy
PSM: Propensity score matching
MELD: Model for end-stage liver disease
EVL: Endoscopic variceal ligation
HR: Hazard ratio
CI: Confidence interval
