# Supplementary material for: Endoscopic variceal obturation and retrograde transvenous obliteration for acute gastric cardiofundal variceal bleeding in liver cirrhosis
Source: BMC Gastroenterol. 2022 Jul 26;22:355. doi: 10.1186/s12876-022-02428-1 (PMC9317208; doi:10.1186/s12876-022-02428-1)
Supplement: Supplementary file 1 — Additional file 1. Supplementary Note. [file 12876_2022_2428_MOESM1_ESM.docx]

**Supplementary Note**

***Endoscopic variceal obturation***

EVO was performed using 21-gauge injection needles. The GVs were punctured and 1–2 mL of a mixture of n-butyl-2-cyanoacrylate (Histoacryl L®; Braun Surgical SA, Rubi, Spain) and Lipiodol Ultra-Fluide (Guerbet, Bois Cedex, France) in a 1:1 ratio was injected, immediately followed by a second injection of 1.0 mL of distilled water to flush the remaining mixture from the needle. During each session, 1–5 mL of mixture was used. Computed tomography scans and follow-up EGD were performed 1 to 3 days after the initial session. If necessary, injections were repeated until the resolution of the GVs was achieved. Follow-up endoscopies were performed 4 weeks later and then at 3-month intervals. Obturation of the GV after EVO was assessed by probing the injected varices with forceps.

***Retrograde transvenous obliteration***

RTOs were performed via a transfemoral or transjugular approach under local anesthesia. An occlusion balloon (Terumo, Tokyo, Japan) was advanced into the shunt in BRTO. Retrograde venography was performed, and collateral veins were embolized using coils (Cook Medical, Bloomington, IN, USA). With the occlusion balloon inflated, sclerosant, a 2:1:2–3 mixture of sodium tetradecyl sulfate foam (Sotradecol; AngioDynamics, Queensbury, NY, USA), Lipiodol Ultra-Fluide, and air was injected into the varix. The occlusion balloon remained inflated after the procedure for 4–20 hours and was removed after stagnation of the sclerosant was confirmed on follow-up radiography (1).

In PARTO, large collateral veins are embolized using coils if present. Amplatzer II vascular plugs (AGA Medical, Golden Valley, Minnesota), ranging from 3 to 22 mm in diameter depending on the diameter of the narrowest part of the shunt as measured on venogram or CT, were deployed. A mixture of hand-cut gelfoam sheets and contrast media was injected through a catheter to embolize the varices and collateral vessels. After embolization of the GVs, the vascular plug was detached and remained as a permanent occlusion of the gastrorenal shunt (2).

**References**

1. Kim DJ, Darcy MD, Mani NB, Park AW, Akinwande O, Ramaswamy RS, Kim SK. Modified Balloon-Occluded Retrograde Transvenous Obliteration (BRTO) Techniques for the Treatment of Gastric Varices: Vascular Plug-Assisted Retrograde Transvenous Obliteration (PARTO)/Coil-Assisted Retrograde Transvenous Obliteration (CARTO)/Balloon-Occluded Antegrade Transvenous Obliteration (BATO). Cardiovasc Intervent Radiol 2018;41:835-847.

2. Gwon DI, Ko GY, Yoon HK, Sung KB, Kim JH, Shin JH, Ko HK, et al. Gastric varices and hepatic encephalopathy: treatment with vascular plug and gelatin sponge-assisted retrograde transvenous obliteration--a primary report. Radiology 2013;268:281-287.

**Supplementary Table 1.** Treatment outcomes according to the type of treatment

| Outcomes | All patients  (n=176) | EVO group (n=90) | RTO group (n=86) | P value |
| --- | --- | --- | --- | --- |
| Bleeding control, n (%) | 171 (97.2) | 88 (97.8) | 83 (96.5) | 0.613 |
| Technical success, n (%) | 176 (100.0) | 90 (100.0) | 86 (100.0) | - |
| Worsening of esophageal varices, n (%) | 36 (20.5) | 12 (14.8) | 24 (27.9) | <0.001 |
| All-variceal rebleeding rate (%) |  |  |  | 0.150 |
| 6 months | 6.1 | 6.1 | 6.1 |  |
| 12 months | 11.1 | 11.2 | 11.1 |  |
| 18 months | 17.9 | 23.4 | 12.9 |  |
| 24 months | 26.3 | 32.4 | 20.8 |  |
| Gastric variceal rebleeding rate (%) |  |  |  | 0.003 |
| 6 months | 4.9 | 6.1 | 3.7 |  |
| 12 months | 8.3 | 11.2 | 5.4 |  |
| 18 months | 15.2 | 23.4 | 7.2 |  |
| 24 months | 22.5 | 32.4 | 12.8 |  |
| Transplantation-free survival rate (%) |  |  |  | 0.597 |
| 6 months | 87.5 | 86.7 | 88.4 |  |
| 12 months | 84.8 | 83.9 | 85.8 |  |
| 18 months | 82.2 | 80.4 | 84.1 |  |
| 24 months | 79.1 | 78.4 | 79.9 |  |

EVO, endoscopic variceal obturation; RTO, retrograde transvenous obliteration.

**Supplementary Table 2.** Baseline characteristics of patients balanced by propensity score matching

|  | All patients  (n=142) | EVO group  (n=71) | RTO group  (n=71) | P value | Standardized difference |
| --- | --- | --- | --- | --- | --- |
| Age | 59.6 ± 11.9 | 59.5 ± 11.9 | 60.0 ± 12.0 | 0.871 | -0.051 |
| Male, n (%) | 101 (71.1) | 54 (76.1) | 47 (66.2) | 0.195 | 0.028 |
| Etiology, n (%) |  |  |  | 0.574 | 0.037 |
| Alcohol | 66 (46.5) | 37 (52.1) | 29 (40.8) |  |  |
| Other | 76 (53.5) | 34 (47.9) | 42 (59.2) |  |  |
| Diabetes, n (%) | 48 (33.8) | 24 (33.8) | 24 (33.8) | 1.000 | 0.010 |
| Hepatocellular carcinoma, n (%) | 44 (31.0) | 21 (29.6) | 23 (32.4) | 0.717 | 0.049 |
| Type of varices, n (%) |  |  |  | 0.863 | 0.010 |
| GOV2 | 87 (61.3) | 44 (62.0) | 43 (60.6) |  |  |
| IGV1 | 55 (38.7) | 27 (38.0) | 28 (39.4) |  |  |
| Size of esophageal varices, n (%) |  |  |  | 0.310 | 0.184 |
| F0-F1 | 80 (56.3) | 37 (52.1) | 43 (60.6) |  |  |
| F2-F3 | 62 (43.7) | 34 (47.9) | 28 (39.4) |  |  |
| Hemoglobin, g/dL | 9.1 ± 2.2 | 9.1 ± 2.4 | 9.1 ± 2.1 | 0.844 | 0.037 |
| Platelet count, × 10^9^/L | 97.5 ± 49.4 | 100.4 ± 47.3 | 95.0 ± 51.8 | 0.493 | 0.185 |
| INR | 1.4 ± 0.3 | 1.4 ± 0.3 | 1.3 ± 0.2 | 0.106 | 0.089 |
| Alanine aminotransferase, IU/L | 38.1 ± 51.5 | 42.0 ± 63.7 | 34.3 ± 35.5 | 0.378 | 0.369 |
| Total bilirubin, mg/dL | 2.9 ± 0.5 | 1.6 ± 1.2 | 1.6 ± 1.1 | 0.784 | 0.780 |
| Serum albumin, g/dL | 1.6 ± 1.1 | 3.0 ± 0.5 | 2.9 ± 0.5 | 0.589 | 0.589 |
| MELD score | 11.9 ± 3.4 | 12.2 ± 3.5 | 11.6 ± 3.3 | 0.277 | 0.185 |
| Beta-blockers, n (%) | 43 (30.3) | 9 (12.7) | 34 (47.9) | <0.001 | -1.100 |

Variables are expressed as mean ± standard deviation or n (%). EVO, endoscopic variceal obturation; RTO, retrograde transvenous obliteration; GOV2, gastroesophageal varices type 2; IGV1, isolated gastric varices type 1; INR, international normalized ratio; MELD, model for end-stage liver disease.

**Supplementary Table 3.** Treatment outcomes of patients balanced by propensity score matching

|  | EVO group (n=71) | RTO group (n=71) | P value |
| --- | --- | --- | --- |
| Bleeding control, n (%) | 71 (100.0) | 69 (97.2) | 0.154 |
| Technical success, n (%) | 71 (100.0) | 71 (100.0) | - |
| Worsening of esophageal varices, n (%) | 11 (15.5) | 23 (32.4) | <0.001 |
| Variceal rebleeding rate (%) |  |  | 0.032 |
| 6 months | 6.0 | 4.5 |  |
| 12 months | 12.1 | 8.3 |  |
| 18 months | 26.4 | 10.5 |  |
| 24 months | 37.3 | 20.0 |  |
| Gastric variceal rebleeding rate (%) |  |  | <0.001 |
| 6 months | 6.0 | 1.6 |  |
| 12 months | 12.1 | 3.6 |  |
| 18 months | 26.4 | 5.8 |  |
| 24 months | 37.3 | 12.5 |  |
| Transplantation-free survival rate (%) |  |  | 0.119 |
| 6 months | 86.7 | 88.4 |  |
| 12 months | 83.9 | 85.8 |  |
| 18 months | 80.4 | 84.1 |  |
| 24 months | 78.4 | 79.9 |  |

EVO, endoscopic variceal obturation; RTO, retrograde transvenous obliteration.
