# Supplementary figures and images for: Endoscopic variceal obturation and retrograde transvenous obliteration for acute gastric cardiofundal variceal bleeding in liver cirrhosis
Source: BMC Gastroenterol. 2022 Jul 26;22:355. doi: 10.1186/s12876-022-02428-1 (PMC9317208; doi:10.1186/s12876-022-02428-1)

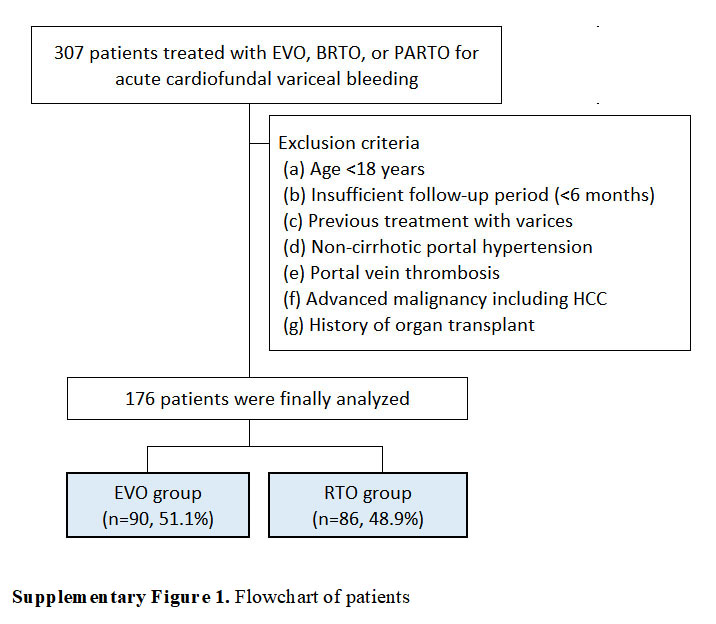

Supplement: Supplementary file 2 — Additional file 2. Supplementary Figure 1. Flowchart of patients. [file 12876_2022_2428_MOESM2_ESM.jpg]
